# The performance of a deep learning system in assisting junior ophthalmologists in diagnosing 13 major fundus diseases: *a prospective multi-center clinical trial*

**DOI:** 10.1038/s41746-023-00991-9

**Published:** 2024-01-11

**Authors:** Bing Li, Huan Chen, Weihong Yu, Ming Zhang, Fang Lu, Jingxue Ma, Yuhua Hao, Xiaorong Li, Bojie Hu, Lijun Shen, Jianbo Mao, Xixi He, Hao Wang, Dayong Ding, Xirong Li, Youxin Chen

**Affiliations:** 1 Department of Ophthalmology, Peking Union Medical College Hospital, Chinese Academy of Medical Sciences, Beijing, China; 2 Key Laboratory of Ocular Fundus Diseases, Chinese Academy of Medical Sciences, Peking Union Medical College, Beijing, China; 3 Department of Ophthalmology, West China Hospital, Sichuan University, Chengdu, China; 4 Department of Ophthalmology, Second Hospital of Hebei Medical University, Shijiazhuang, China; 5 Department of Retina, Tianjin Medical University Eye Hospital, Tianjin, China; 6 Department of Retina Center, Affiliated Eye Hospital of Wenzhou Medical University, Hangzhou, Zhejiang Province China; 7 School of Information Science and Technology, North China University of Technology, Beijing, China; 8 Beijing Key Laboratory on Integration and Analysis of Large-scale Stream Data, Beijing, China; 9 Visionary Intelligence Ltd., Beijing, China; 10 MoE Key Lab of DEKE, Renmin University of China, Beijing, China

**Keywords:** Population screening, Adaptive clinical trial

## Abstract

Artificial intelligence (AI)-based diagnostic systems have been reported to improve fundus disease screening in previous studies. This multicenter prospective self-controlled clinical trial aims to evaluate the diagnostic performance of a deep learning system (DLS) in assisting junior ophthalmologists in detecting 13 major fundus diseases. A total of 1493 fundus images from 748 patients were prospectively collected from five tertiary hospitals in China. Nine junior ophthalmologists were trained and annotated the images with or without the suggestions proposed by the DLS. The diagnostic performance was evaluated among three groups: DLS-assisted junior ophthalmologist group (test group), junior ophthalmologist group (control group) and DLS group. The diagnostic consistency was 84.9% (95%CI, 83.0% ~ 86.9%), 72.9% (95%CI, 70.3% ~ 75.6%) and 85.5% (95%CI, 83.5% ~ 87.4%) in the test group, control group and DLS group, respectively. With the help of the proposed DLS, the diagnostic consistency of junior ophthalmologists improved by approximately 12% (95% CI, 9.1% ~ 14.9%) with statistical significance (*P* < 0.001). For the detection of 13 diseases, the test group achieved significant higher sensitivities (72.2% ~ 100.0%) and comparable specificities (90.8% ~ 98.7%) comparing with the control group (sensitivities, 50% ~ 100%; specificities 96.7 ~ 99.8%). The DLS group presented similar performance to the test group in the detection of any fundus abnormality (sensitivity, 95.7%; specificity, 87.2%) and each of the 13 diseases (sensitivity, 83.3% ~ 100.0%; specificity, 89.0 ~ 98.0%). The proposed DLS provided a novel approach for the automatic detection of 13 major fundus diseases with high diagnostic consistency and assisted to improve the performance of junior ophthalmologists, resulting especially in reducing the risk of missed diagnoses. ClinicalTrials.gov NCT04723160

## Introduction

Fundus diseases have become the most common irreversible leading causes of blindness worldwide^1^. With the increase in the aging population and life expectancy, the prevalence of major blindness-leading eye diseases is steadily increasing as well. According to epidemiological surveys, it is projected that within the next 20 years, the population with age-related macular degeneration (AMD) will reach 288 million^2^, approximately 600 million people will have diabetes, with 34.6% expected to have diabetic retinopathy (DR)^3^, and glaucoma will affect 111.8 million individuals^4^. For the abovementioned and other major fundus diseases, early detection, timely referral and treatment are the key strategies for blindness prevention. However, conventional screening is time-consuming and costly and calls for large numbers of human assessors and sustained financial assistance, both of which still remain as huge challenges globally. Furthermore, the shortage of experienced ophthalmologists limits the screening process in underdeveloped places.

Deep learning (DL) is an important subfield of artificial intelligence (AI), which can extract underlying features in big data from multiple processing layers using convolutional neural networks (CNNs) and then identify images and features^5^. Since the diagnosis of ophthalmic diseases is highly dependent on image recognition, the specialty of ophthalmology has become particularly well suited to DL techniques and their real-world application^6^. Over the last decades, advances in DL technology have allowed the automatic identification of various ophthalmological diseases from fundus images, especially major blindness-leading diseases, including DR^7–9^, AMD^10,11^, glaucoma^12–14^, and retinopathy of prematurity (ROP)^15,16^. In addition, some studies have also proven the feasibility of DL system (DLS) in detecting multiple fundus diseases or lesions simultaneously, including studies from our group, which indicates the possible application of DLS for large-scale screening of multiple fundus diseases in the future^17–20^. We should take a more prudent approach to this newly sprouted methodology and carefully explore its performance and value in practical applications through prospective clinical trials.

In our last report, we developed a DLS that could detect 12 fundus diseases using retrospectively collected datasets with over 60000 fundus images. We then further upgraded the model to identify more diseases through training and validation using more fundus images. This study, by means of a prospective clinical trial, aims to evaluate both the diagnostic performance and the potential value of a DLS in assisting junior ophthalmologists in the detection of 13 major fundus diseases, namely referable DR, retinal vein occlusion (RVO), retinal artery occlusion (RAO), pathologic myopia, retinal detachment (RD), retinitis pigmentosa (RP), atrophic and neovascular age-related macular degeneration (AMD), epiretinal membrane (ERM), macula hole (MH), central serous chorioretinopathy (CSC), suspect glaucomatous optic neuropathy (GON), and optic nerve atrophy.

## Results

### Study population

**Fig. 1:**
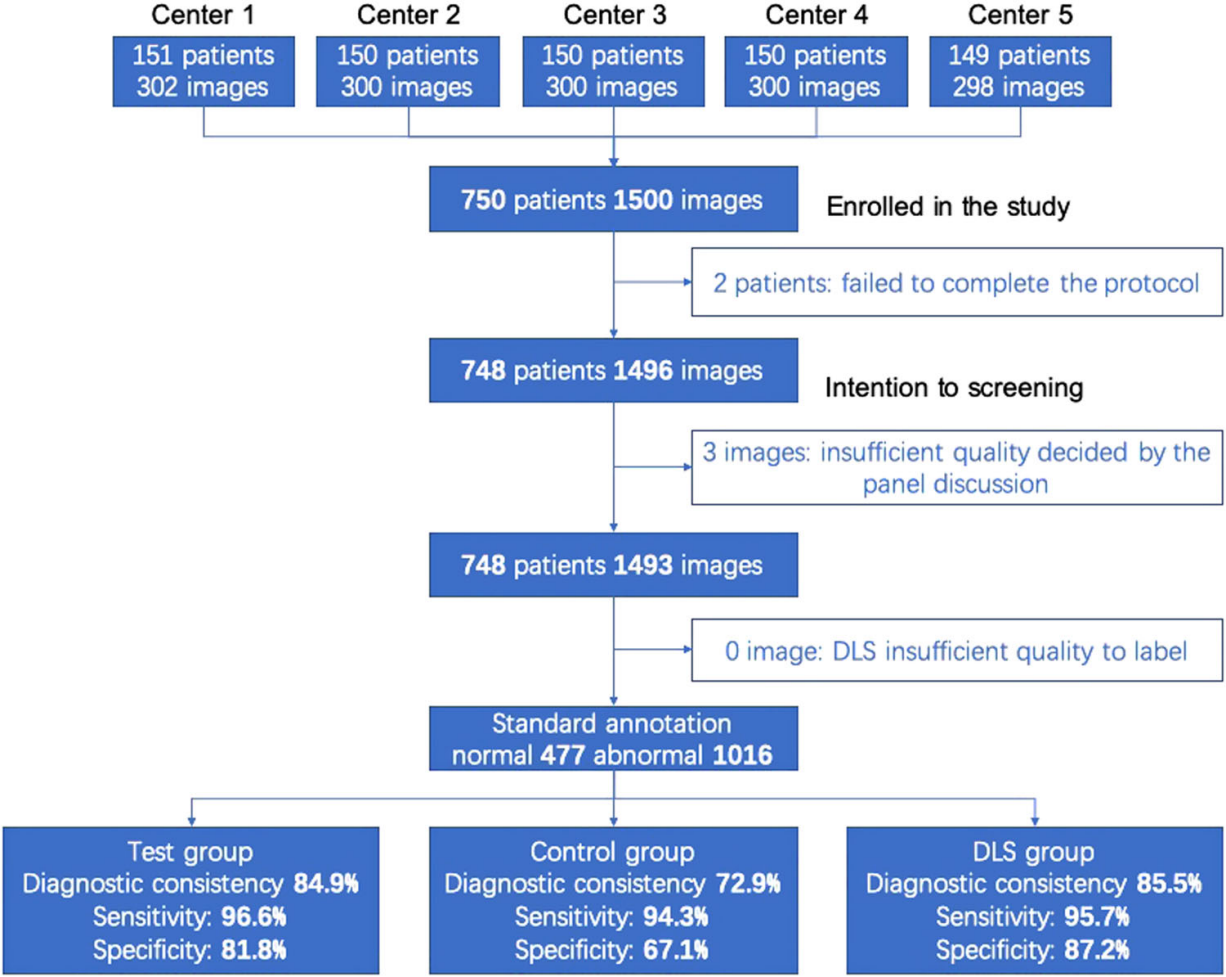
Flow diagram showing the final disposition of each participant in the enrolled and fully analyzable populations. Sensitivity and specificity refer to the performance in detecting any fundus disease. Center 1: Peking Union Medical College Hospital, Center 2: West China Hospital Sichuan University, Center 3: The Second Hospital of Hebei Medical University, Center 4: Tianjin Medical University Eye Hospital, Center 5: The Eye Hospital of Wenzhou Medical University.

A total of 750 patients were screened from five participating hospitals, and 748 of them completed all procedures. After the standard annotation, three images were excluded due to unsatisfactory image quality. A subset of 1493 images could be fully analyzed (Fig. 1). The mean (standard deviation, SD) age of the patients was 51.7 (14.7) years (range, 18 ~ 75), and 324 (43.3%) of them were male. All participants were Chinese Han patients. Regarding the medical history of the patients, 152 (20.3%), 216 (28.9%) and 104 (13.9%) patients reported having diabetic mellites, hypertension and hyperlipemia, respectively. The fundus cameras used in this study were Kowa Nonmyd 7 (206, 13.8%), Visucam 224 (Carl Zeiss Meditec) (300, 20.1%) and Canon CR2 (990, 66.2%).

According to the annotations and discussions of the six senior ophthalmologists, 477 (32.0%) images were labeled with normal fundus. The other 1016 (68.1%) images were labeled with diseases, of which, 1386 (92.8%) images were labeled with one disease, 78 (5.2%) and 29 (1.9%) images were labeled with two and three diseases, respectively. A total of 251 (16.8%) images were annotated with “other abnormalities” as the only label, which referred to the abnormalities not included in this study.

### DLS characteristics

The diagnostic consistency was 84.9% (1268/1493, [95% confidence interval (CI), 83.0% ~ 86.9%]), 72.9% (1089/1493, [95% CI, 70.3% ~ 75.6%]) and 85.5% (1276/1493, [95% CI, 83.5% ~ 87.4%]) in the test group, control group and DLS group, respectively. With the help of the proposed DLS, the diagnostic consistency of the junior ophthalmologists significantly improved by approximately 12% (95% CI, 9.1% ~ 14.9%; *P* < 0.001). The overall abnormality diagnostic sensitivity values of the test group, control group and DLS group were 96.6%, 94.3% and 95.7%, respectively, with the corresponding specificities of 81.8%, 67.1% and 87.2%. The diagnostic accuracy in the test group, control group and DLS group was 44.2% (660/1493), 60.5% (903/1493) and 40.0% (597/1493), respectively. The accuracy of the principal diagnosis was 72.6% (1084/1493) and 68.3% (1020/1493) in the test group and control group, respectively.

In the detection of the 13 diseases, the test group achieved significantly higher sensitivities (72.2% ~ 100.0%, median 91.7%) than and comparable specificities (90.8% ~ 98.7%, median 97.5%) to the control group (sensitivities, 50.0% ~ 100.0%, median 75.9%; specificities 96.7 ~ 99.7%, median 98.3%). With the assistance of the proposed DLS, the diagnostic sensitivity of the junior ophthalmologists in the detection of specific diseases was greatly improved. The DLS group presented similar performance to the test group in the detection of any fundus abnormality (sensitivity, 87.2%; specificity, 95.7%) and of each of the 13 diseases (sensitivity, 83.3% ~ 100.0%, median 94.0%; specificity, 89.0 ~ 98.0%, median 95.3%). The mean F1 scores of the test group, control group and DLS group was 61.6% ± 16.8% (range 36.4% ~ 94.2%), 65.4% ± 16.7% (range 27.1% ~ 89.94%) and 54.4% ± 19.1% (range 31.3% ~ 94.9), respectively. (Table 1)

**Table 1:** The sensitivity, specificity and F1 score on the diagnosis of different diseases in the three groups.

	N	Test group (value [95% CI])			Control group (value [95% CI])			DLS group (value [95% CI])		
		Sensitivity	Specificity	F1 score	Sensitivity	Specificity	F1 score	Sensitivity	Specificity	F1 score
Normal fundus	477	96.57% [95.45%, 97.68%]	81.76% [78.30%, 85.23%]	94.17% [92.73%, 95.61%]	94.31% [92.89%, 95.73%]	67.09% [62.87%, 71.30%]	89.94% [88.09%, 91.79%]	95.68% [94.43%, 96.93%]	87.21% [84.21%, 90.21%]	94.89% [93.54%, 96.24%]
Referable DR	165	95.15% [91.87%, 98.43%]	93.52% [92.20%, 94.85%]	76.96% [70.54%, 83.39%]	87.27% [82.19%, 92.36%]	97.29% [96.42%, 98.16%]	83.48% [77.81%, 89.14%]	96.36% [93.51%, 99.22%]	88.33% [86.60%, 90.06%]	66.39% [59.18%, 73.60%]
RAO	137	87.50% [61.65%, 98.45%]	97.70% [96.93%, 98.46%]	43.75% [19.45%, 68.06%]	62.50% [38.78%, 86.22%]	99.39% [98.99%, 99.79%]	57.14% [32.89%, 81.39%]	87.50% [61.65%, 98.45%]	96.41% [95.46%, 97.36%]	33.74% [10.57%, 56.91%]
RVO	16	89.13% [83.94%, 94.32%]	97.49% [96.66%, 98.32%]	83.39% [77.15%, 89.62%]	76.09% [68.97%, 83.20%]	98.60% [97.97%, 99.22%]	80.16% [73.48%, 86.83%]	91.30% [86.60%, 96.01%]	96.24% [95.22%, 97.25%]	80.00% [73.30%, 86.70%]
Pathologic myopia	118	100.00% [96.92%, 100.0%]	97.82% [97.05%, 98.59%]	88.72% [83.01%, 94.43%]	94.92% [90.95%, 98.88%]	97.02% [96.12%, 97.92%]	82.66% [75.83%, 89.49%]	98.31% [94.01%, 99.79%]	97.60% [96.79%, 98.41%]	86.89% [80.80%, 92.98%]
RD	11	100.00% [71.51%, 100.0%]	98.72% [98.15%, 99.29%]	53.66% [24.19%, 83.13%]	81.82% [48.22%, 97.72%]	99.73% [99.31%, 99.93%]	75.00% [49.41%, 100.00%]	90.91% [58.72%, 99.77%]	97.98% [97.26%, 98.69%]	39.22% [10.36%, 68.07%]
RP	25	100.00% [86.28%, 100.0%]	98.50% [97.88%, 99.12%]	69.44% [51.39%, 87.50%]	84.00% [63.92%, 95.46%]	99.46% [99.08%, 99.83%]	77.78% [61.48%, 94.07%]	96.00% [79.65%, 99.90%]	97.55% [96.76%, 98.34%]	56.47% [37.04%, 75.91%]
CSC	36	91.67% [77.53%, 98.25%]	95.81% [94.78%, 96.84%]	50.77% [34.44%, 67.10%]	61.11% [45.19%, 77.04%]	98.28% [97.62%, 98.95%]	53.01% [36.71%, 69.32%]	100.00% [90.26%, 100.0.%]	92.79% [91.47%, 94.12%]	40.68% [24.63%, 56.72%]
MH	39	93.10% [77.23%, 99.15%]	97.95% [97.23%, 98.68%]	62.79% [45.20%, 80.38%]	100.00% [88.06%, 100.0%]	98.50% [97.87%, 99.12%]	72.50% [56.25%, 88.75%]	93.10% [77.23%, 99.15%]	95.08% [93.97%, 96.19%]	42.18% [24.21%, 60.16%]
ERM	84	96.39% [89.80%, 99.25%]	91.42% [89.96%, 92.88%]	56.34% [45.73%, 66.94%]	75.90% [66.70%, 85.10%]	97.59% [96.79%, 98.39%]	70.00% [60.20%, 79.80%]	93.98% [86.50%, 98.02%]	88.23% [86.54%, 89.91%]	47.71% [37.03%, 58.39%]
Neovascular AMD	54	72.22% [60.28%, 84.17%]	97.71% [96.93%, 98.48%]	61.91% [48.95%, 74.86%]	64.81% [52.08%, 77.55%]	98.33% [97.67%, 98.99%]	61.94% [48.99%, 74.89%]	83.33% [73.39%, 93.27%]	97.01% [96.13%, 97.89%]	63.38% [50.53%, 76.23%]
Atrophic AMD	77	85.71% [77.90%, 93.53%]	90.75% [89.24%, 92.26%]	48.17% [37.01%, 59.33%]	50.65% [39.48%, 61.82%]	96.68% [95.75%, 97.61%]	47.85% [36.70%, 59.01%]	89.61% [82.80%, 96.43%]	88.98% [87.35%, 90.61%]	45.70% [34.57%, 56.83%]
Suspect GON	36	88.89% [73.94%, 96.89%]	92.59% [91.24%, 93.93%]	36.37% [20.65%, 52.08%]	50.00% [33.67%, 66.33%]	94.58% [93.42%, 95.74%]	27.07% [12.56%, 41.59%]	94.44% [81.34%, 99.32%]	89.91% [88.36%, 91.46%]	31.33% [16.18%, 46.48%]
Optic nerve atrophy	34	91.18% [76.32%, 98.14%]	96.02% [95.02%, 97.03%]	50.41% [33.60%, 67.21%]	55.88% [39.19%, 72.57%]	98.15% [97.46%, 98.84%]	47.50% [30.71%, 64.28%]	94.12% [80.32%, 99.28%]	95.27% [94.18%, 96.36%]	47.40% [30.62%, 64.19%]
Other abnormality	330	61.89% [56.63%, 67.15%]	71.07% [68.47%, 73.68%]	46.77% [41.39%, 52.16%]	57.62% [52.27%, 62.97%]	85.41% [83.38%, 87.43%]	55.02% [49.66%, 60.39%]	49.39% [43.98%, 54.80%]	73.56% [71.03%, 76.09%]	40.60% [35.30%, 45.90%]

### Post hoc analysis

#### Doctors’ behaviors with DLS assistance

**Fig. 2:**
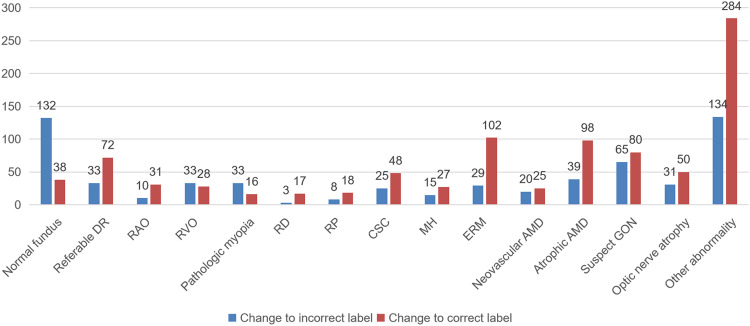
The situation of the doctors who changed their decision in each category. For most diseases, the DLS could help junior ophthalmologists make the correct diagnostic decision.

Compared with the control group, the junior ophthalmologists changed their annotations in 888 (59.4%) images. Among them, 801 (53.5%) images were changed according to the AI’s suggestion. Although in some cases, the junior ophthalmologists changed their diagnostic decision from a correct to an incorrect label according to the suggestion of the DLS, they were assisted to correct incorrect labels more often, as Fig. 2 indicates.

**Fig. 3:**
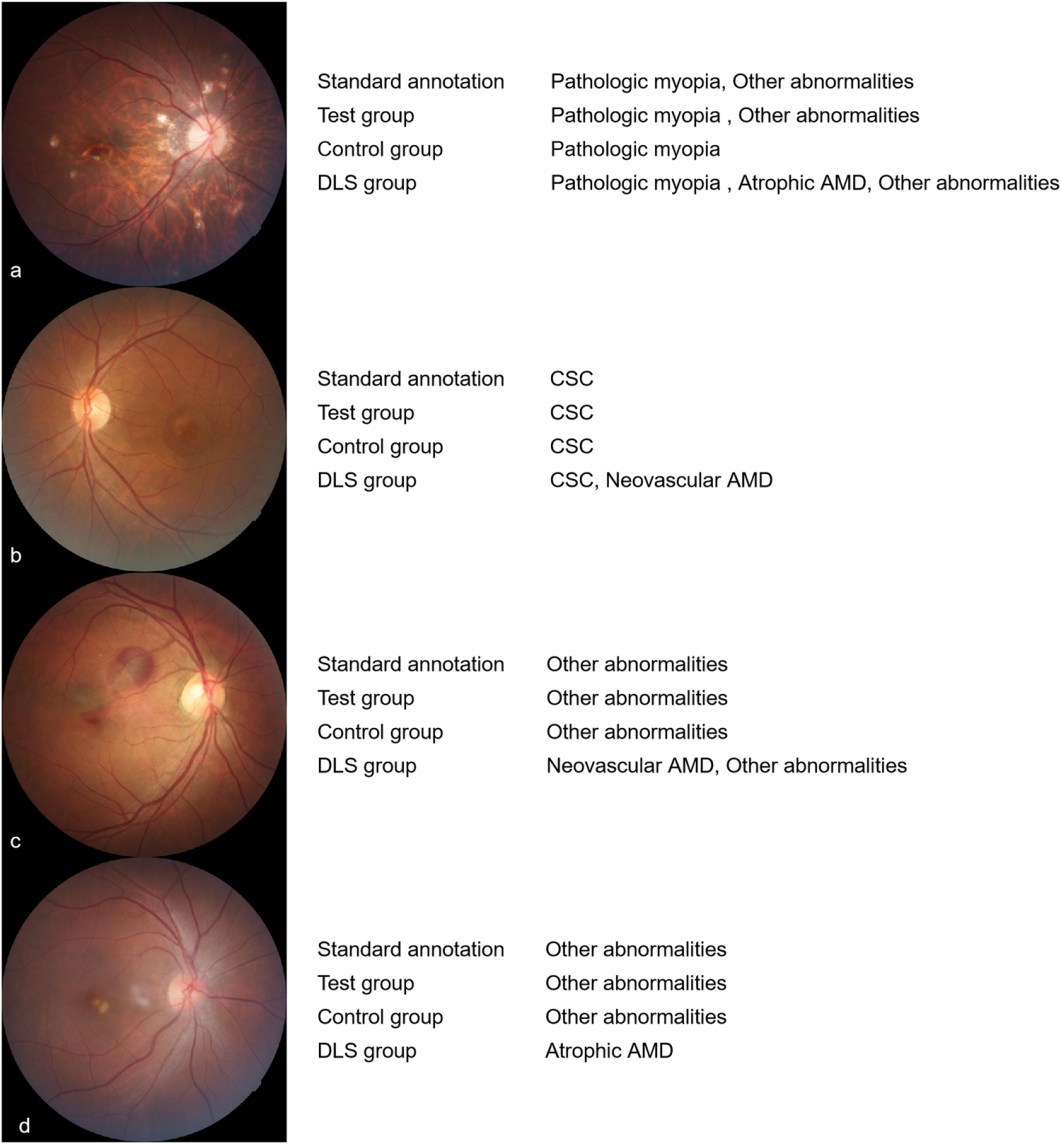
Cases of junior ophthalmologists who overruled the DLS’s suggestions. Case **a**, the doctor changed the diagnosis: accepted the suggestion of “other abnormalities” but overruled “atrophic AMD”. Cases **b** and **c**, the doctors overruled the DLS’s additional suggestions and did not change their diagnosis. Case **d**: the doctor insisted the diagnosis despite of the DLS’s suggestion.

The conditions that junior ophthalmologists overruled DLS’s suggestion mostly occurred when the image were not so typical or contained some similar features of different diseases, which seemed difficult for the DLS to distinguish but obvious for junior ophthalmologists to identify. In general, junior ophthalmologists intended to accept the DLS’s suggestions but also help to correct DLS’s error in special cases (Fig. 3).

We also reviewed the images labeled with “other abnormalities”. This category in our study contained various diseases. Some of them could be identified through fundus image directly, including punctate inner choroidopathy (PIC). Most of them presented with some lesions that were not so typical and needed further clinical inspections for diagnosis, including macular atrophy, subretinal proliferation and some exudative lesions.

#### Two-step protocol

The diagnostic consistency, accuracy and accuracy of the principal diagnosis of the two-step protocol were 86.9%, 46.0% and 74.6%, respectively. Among the three metrics, the two-step protocol achieved higher diagnostic consistency and accuracy of the principal diagnosis than the test group and DLS group (Table 2). Relative to the test group, the two-step protocol achieved similar sensitivities, specificities and F1 score for the 13 diseases. These results indicate that this two-step protocol could be considered another efficient way to screen fundus imaging, helping to reduce manpower burden.

**Table 2:** The sensitivity, specificity and F1 score of the diagnosis of different diseases in the “two-step” protocol.

	Sensitivity (value [95% CI])	Specificity (value [95% CI])	F1 (value [95% CI])
Normal fundus	89.73% [86.65%, 92.30%]	95.47% [94.01%, 96.67%]	95.33% [94.04%,96.63%]
Referable DR	95.15% [90.67%, 97.88%]	93.52% [92.06%, 94.79%]	76.96% [71.56%,82.24%]
RAO	81.25% [54.35%, 95.95%]	97.70% [96.80%, 98.40%]	41.27% [24.00%,57.60%]
RVO	90.51% [84.32%, 94.85%]	97.57% [96.60%, 98.32%]	84.35% [78.57%,90.11%]
Pathologic myopia	98.31% [94.01%, 99.79%]	98.04% [97.16%, 98.70%]	88.89% [84.01%,93.35%]
RD	90.91% [58.72%, 99.77%]	98.65% [97.92%, 99.17%]	48.78% [26.93%,66.85%]
RP	100.00% [86.28%, 100.00%]	98.50% [97.74%, 99.06%]	69.44% [56.04%,80.57%]
CSC	91.67% [77.53%, 98.25%]	96.02% [94.88%, 96.96%]	51.97% [40.00%,63.15%]
MH	93.10% [77.23%, 99.15%]	97.95% [97.09%, 98.61%]	62.79% [48.80%,75.26%]
ERM	94.05% [86.65%, 98.04%]	91.41% [89.83%, 92.82%]	55.63% [47.85%,63.09%]
Neovascular AMD	72.22% [58.36%, 83.54%]	97.71% [96.79%, 98.42%]	61.90% [49.96%,73.78%]
Atrophic AMD	84.42% [74.36%, 91.68%]	90.89% [89.27%, 92.34%]	47.97% [39.74%,56.00%]
Suspect GON	88.89% [73.94%, 96.89%]	93.07% [91.64%, 94.32%]	37.87% [27.68%,47.61%]
Optic nerve atrophy	91.18% [76.32%, 98.14%]	96.09% [94.97%, 97.03%]	50.82% [38.57%,62.27%]
Other abnormality	61.21% [55.72%, 66.50%]	72.74% [70.09%, 75.28%]	47.59% [42.86%,52.30%]

## Discussion

To the best of our knowledge, this is the first clinical trial to prove that a DLS could help junior ophthalmologists to attain significant improvement in diagnostic consistency on the detection of multiple major fundus diseases. We also proved that the novel DLS-assisted junior ophthalmologists-based image reading mode for multiple fundus diseases screening is an effective method for clinical application. To overcome the shortage of medical resources and ophthalmic specialists, the DLS could also work effectively when applied either independently or as an initial filter. Since there is no other clinical trials of multiple fundus diseases diagnostic systems reported, we used the AI-based DR grading system for reference. In the pivot trial of an autonomous AI-based diagnostic system for the detection of DR, the prespecified primary endpoints of sensitivity and specificity were set to exceed 85% and 82.5%, respectively^21^. In our study, the overall disease diagnostic sensitivity and specificity in the DLS reached 96.6% and 81.8%, respectively. Additionally, sensitivity values exceeding 85% were achieved in 12 diseases in the test group and DLS group, and all specificity values exceeded 82.5% (88.2% ~ 98.0%). With its high sensitivity, the DLS could help to detect specific fundus diseases effectively by identifying fundus abnormalities and assist in the referral of patients for specialized investigation and evaluation. Furthermore, the high specificity could help to prevent unnecessary referrals and medical costs. In addition to the good diagnostic performance of the proposed DLS, a prospectively collected dataset also provided a real spectrum of the selected diseases in clinical practice. In addition to the selected 13 major fundus diseases, we also improved the classification with a new category of “other fundus abnormalities”, allowing the DLS to classify abnormalities that were not defined in the study and make the detection more reasonable and meaningful. As we described in our previous work^20^, the DLS was developed as a multi-label model to simultaneously classify abnormal versus normal fundus images and to accurately detect the presence of multiple fundus diseases. Considering that the images could have more than one label, we introduced diagnostic consistency as a new indicator. Instead of the evaluation of each label, we provide a new way to evaluate the performance on each image. Since the report of the whole image is what would be finally sent to the patients, reliable results regarding the image instead of each label should be valued as more important and meaningful. We considered that patients should be referred to the hospital when any of the diseases are identified. Therefore, the model should be able to detect at least one of the existing diseases, consistent with the original intention in defining the new indicator.

In addition to evaluating the proposed DLS, another important contribution of this work was that we also explored its potential value for improving the diagnostic level of junior ophthalmologists. The results showed that with the assistance of the DLS, the diagnostic consistency of junior ophthalmologists increased 12% (from 72.9% to 84.9%). In this study, the test group and DLS group achieved similar diagnostic consistency, which suggested that the DLS alone could work sufficiently. However, it is still crucial to have human doctors involved. Although AI-based disease screening and recognition has been widely explored and applied in real clinical practice, it also introduces associated risks including misdiagnosis. In the present stage, AI systems still cannot be considered as “moral agents”^22^ or therefore “responsible entities”^23^. It is not just a technological issue, but a sociotechnical problem. To the best of our knowledge, this is also the first report to explore this new DLS-assisted diagnostic model, which not only helps to improve the diagnostic level of junior ophthalmologists, but also ensures that it can be used under the supervision of qualified ophthalmologists. We also noticed that the sensitivity for any fundus disease was comparable between the test group and control group, but regarding the sensitivity for each disease, the test group was significantly better than the control group, especially for certain diseases, including suspect GON, optic nerve atrophy, atrophic AMD and CSC. These results indicated that junior ophthalmologists could distinguish abnormalities from normal fundus but still miss the diagnosis of specific diseases, a shortcoming that could be addressed with DLS assistance. Thus, the omission diagnostic rate would be greatly decreased, as would the restriction of junior ophthalmologists’ specialization for the diagnosis of fundus diseases. However, the diagnostic accuracy of the control group (60.5%) was higher than that of the test group (44.2%). We reviewed the annotation and found that the DLS tended to annotate more labels for one image. This will assist junior ophthalmologists in reducing missed diagnosis but also affect the diagnostic accuracy at the same time. Moreover, in the post hoc analysis, the data indicated that the junior ophthalmologists tended to trust the DLS and change the annotation according to the AI’s suggestions. This phenomenon provides us with a new insight into the reactions of doctors to DLSs.

Deep learning-assisted diagnosis has been widely used in the field of ophthalmology for image recognition with the objective of overcoming the shortage of specialized medical services and inexperienced ophthalmologists^24^. After successful attempts to detect a single fundus disease, DLSs have also recently been proven to be able to recognize multiple (more than 10) diseases recently^17,18,20,25–27^. Most of the studies successfully yielded satisfactory indicators. Son et al.^17^ reported successful automatic detection of 12 fundus abnormalities using DLSs developed by DR datasets and achieved high sensitivity and specificity. Some of the fundus lesions or abnormalities could directly indicate a specific disease, while other lesions may reflect the same disease. For example, retinal hemorrhage, microaneurysm and cotton wool spots could occur together in diabetic retinopathy. Cen LP et al.^18^ reported a new DLS for the detection of 39 fundus diseases or conditions and provided referral decisions. According to their designation, the model reports all positive labels for each image. However, some of the labels overlapped. A report with complicated diseases and lesions would lead to unnecessary confusion and excessive referral. Recently, the AI-assisted screening of 10 retinal and optic nerve diseases as reported by Dong L et al.^26^ achieved a sensitivity of 89.8% in detecting any of the 10 retinal diseases. Lin D et al.^27^ reported a DLS for the detection of 14 common fundus diseases that achieved a mean sensitivity of 89.7% during a successful application in real clinical practice. Both models were trained and validated using large datasets and achieved satisfactory results. The sensitivity values were slightly lower than those in our study mainly because we set an additional category of other abnormalities for the classification of the diseases not selected in the study. However, neither of the two studies investigated the real clinical practice workflow. In addition to evaluating the proposed DLS itself, we also explore its value in improving the diagnostic capabilities of the junior ophthalmologists, which indicated a novel clinical application model for the collaboration of doctors and DLS.

From our perspective, as a screening tool primarily used in remote regions lacking specialized ophthalmic services, DLSs designed to directly detect fundus diseases are more straightforward to understand and interpret than those focusing on fundus abnormalities or lesions. In this study, we selected specific diseases, mostly comprising those that act as leading causes of blindness and that need early detection and intervention. Some rare or complicated diseases, including rare inherited retinal or macular degeneration and uveitis, were not listed to avoid a small sample size for training and misleading reports during the screening process while satisfying considerations of effectiveness and cost-effectiveness. They would be classified into “other abnormalities” with the intention that a final diagnosis would be rendered after further evaluation by specialists. As we mentioned above, the images in the category of “other abnormalities” are mostly need more clinical inspections for diagnosis, this will not bring additional referral burden in clinical practice. Some retinal diseases that are mostly located at the peripheral retina, such as peripheral retinal breaks or degeneration, were also not included considering the limited scope of color fundus images.

This study has some limitations. First, although the dataset represented the true spectrum of the selected fundus diseases, some of the categories contained a few images and might result in bias in the results. All participants in this study were Chinese Han patients. Further enlargement of the prospective datasets including more patients and more ethnicities is still needed in the future work. Second, some diseases selected in this study started from peripheral retinal area which is beyond the scope of the fundus image such as RD and RP. Therefore, the DLS could not detect them at the initial stage. With the adoption of widefield color fundus photography, the problem could be solved to a certain extent. Third, since the junior ophthalmologists’ diagnostic capacity obtained a great improvement with the assistance of the DLS, this technology could be used for educational purpose as another application scenario. This is also a meaningful topic and needs more comprehensive investigation and evaluation in the future work.

In this prospective clinical trial, we first proved that with the assistance of DLS, junior ophthalmologists achieved significant improvement in the diagnostic consistency and sensitivity for 13 major fundus diseases. The system could also work effectively when applied independently as an automatic screening tool or as an initial filter to reduce manpower burden. The DLS-assisted junior ophthalmologist image reading mode could be considered a feasible method for implementation in clinical practice.

## Methods

### Autonomous AI diagnostic system

**Fig. 4:**
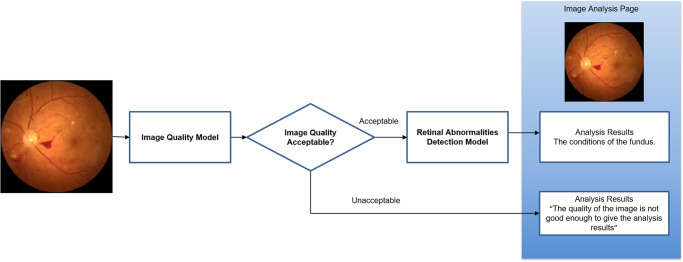
Workflow of the proposed DLS. The DLS was consisted of a quality assessment model and a diagnostic model.

The proposed DLS consists of two components: an image quality assessment model and a diagnostic model. The system workflow is shown in Fig. 4. The DLS takes a single fundus image as input, then starts with the image quality assessment model, which determines whether the image quality is suitable for diagnosis. If so, the diagnostic model will generate diagnostic suggestions; otherwise, the system issues an alert, indicating that the image quality is not suitable for diagnosis and that the system cannot provide diagnostic suggestions.

**Fig. 5:**
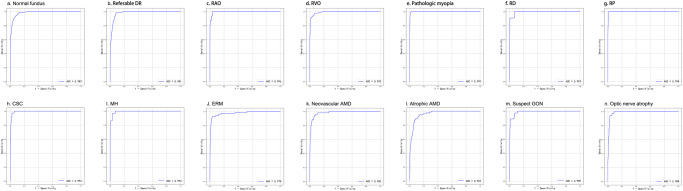
The performance of the proposed DLS in the test group. The ROC curves and AUC values of normal fundus and 13 diseases in the test set of the proposed DLS are listed. The AUC value of each category could reach over 96%.

The diagnostic model used in this system is an extension of the model in our previous work^20^. The fundus disease diagnosis model is based on a CNN model, seResNext50, as the main structure. The fully connected layer is designed with two branches for determining whether there is a disease and which specific diseases are present. We trained three parallel seResNext50 models with late fusion to better stabilize the prediction results. In terms of the dataset, with respect to our previous work, the system collects more color fundus photography data, expanding the total data volume to 81 395 images (training set 77 181, validation set 1087, and test set 3127). Compared with the model in our previous work, the model used in this system identifies two additional diseases, CSC and RAO, but no longer detects papilledema, mainly because papilledema is not a single disease but rather a lesion in several diseases. In the test set, the average sensitivity for all diseases was 89.9%, and the average specificity was 95.3%. The receiver operator characteristic (ROC) curve and area under the curve (AUC) value for each disease in the test set are shown in Fig. 5.

The image quality model used in the system is a regression model built on top of the ResNet-34 CNN. The dataset for this model consists of 31082 fundus color images (25082 for the training set and 3000 each for the validation and test sets). The model produces a real-valued score as an assessment of image quality, which the system uses as input to determine whether it meets an acceptable quality threshold. In the test set, the model achieved a sensitivity of 92.9% and a specificity of 92.4%.

### Study design

This study was conducted by five tertiary hospitals in different parts of China, including Peking Union Medical College Hospital, West China Hospital Sichuan University, the Second Hospital of Hebei Medical University, Tianjin Medical University Eye Hospital, and the Eye Hospital of Wenzhou Medical University. A total of 750 participants were prospectively screened in this observational study at the five centers from August 2020 to January 2021. Six expert ophthalmologists (associated professors for at least five years) were invited to make standard annotations for the images. Nine junior ophthalmologists (residents or attending physicians with less than three years of seniority) were trained and read the images for assessment in the study. The current study complied with the Declaration of Helsinki and was approved by the ethics committee of each center, and all participants provided written informed consent. It is registered on ClinicalTrials.gov with registration number NCT04723160.

### Study subjects

The target population comprised outpatients aged 18 ~ 75 years who visited one of the five participating hospitals and underwent fundus imaging for medical necessities. They were documented with complete medical records and received a comprehensive ophthalmic examination. The fundus images were taken with a (non-) mydriasis fundus camera in both eyes by skilled technicians applying a standard operating procedure to assure the image quality for the subsequent annotation and reading. Those patients from whom clear fundus images in either eye could not be obtained or who were considered unsuitable to participate in the study were excluded. The (non-) mydriasis status of the patients was not needed and thus discarded. The operators took the fundus images according to a standardized imaging protocol, which required capturing fovea-centered 55° fundus images per eye containing the whole posterior pole. The inclusion and exclusion criteria for the patients and images are described in Table 3.

**Table 3:** The inclusion and exclusion criteria for the participating patients and fundus images.

	Inclusion criteria	Exclusion criteria
Patients	·age: 18 ~ 75 years ·fundus photographs taken for medical purposes ·ability to yield clear fundus images in both eyes	• unable to understand the study • unable to or unwillingness to sign the informed consent • unable to get clear fundus images in either eyes • other reasons for unsuitability as determined by the assessors
Images	• the images could be taken with or without mydriasis; • the images should contain the whole posterior pole of the fundus including the optic disc, macular and the vascular arc.	• lack of clarity due to opaqueness of the refracting media • poor quality including loss of focus, artifacts, underexposure or overexposure.

### Fundus diseases

The selection of diseases was decided according to their prevalence and the threat to visual function, also accounting for their clinical potential for screening using fundus images. We selected 13 major fundus diseases with standard diagnostic criteria, including referable DR, RVO, RAO, pathologic myopia, RD, RP, atrophic and neovascular AMD, ERM, MH, CSC, suspect GON, and optic nerve atrophy. If the images contained signs of other diseases that were not included in the selected 13 diseases, they were classified into the category of “other fundus abnormalities”. Considering the potentially different treatments and prognoses of atrophic and neovascular AMD, these conditions were labeled as two diseases in this study. The definition and diagnostic criteria are listed in the Supplementary Information (Supplementary Table 1).

### Reading protocol and study groups

The fundus images were collected and deidentified for reading. In this study, the images could be labeled with at most three diseases to identify the existing diseases. If more than one disease was labeled out in a single image, the diseases were listed in order of their importance and urgency for referral. The one listed first was selected as the “principal diagnosis”.

A panel of six expert ophthalmologists was invited to label the images, forming the standard annotation. All experts were ophthalmic specialists who had been working as retina specialists for at least five years. Five of them were annotation experts and were thus assigned to make the annotations. The other expert served as an arbitrator who was assigned to arbitrate and lead the expert panel discussion. Each included image was annotated once by each annotation expert. Labels that were consistent among at least three experts were retained directly. Otherwise, they were resolved in a panel discussion involving all six experts. If an agreement could still not be reached in the panel discussion, the final label was decided by the arbitration expert, who had the longest working experience as a retinal specialist.

**Fig. 6:**
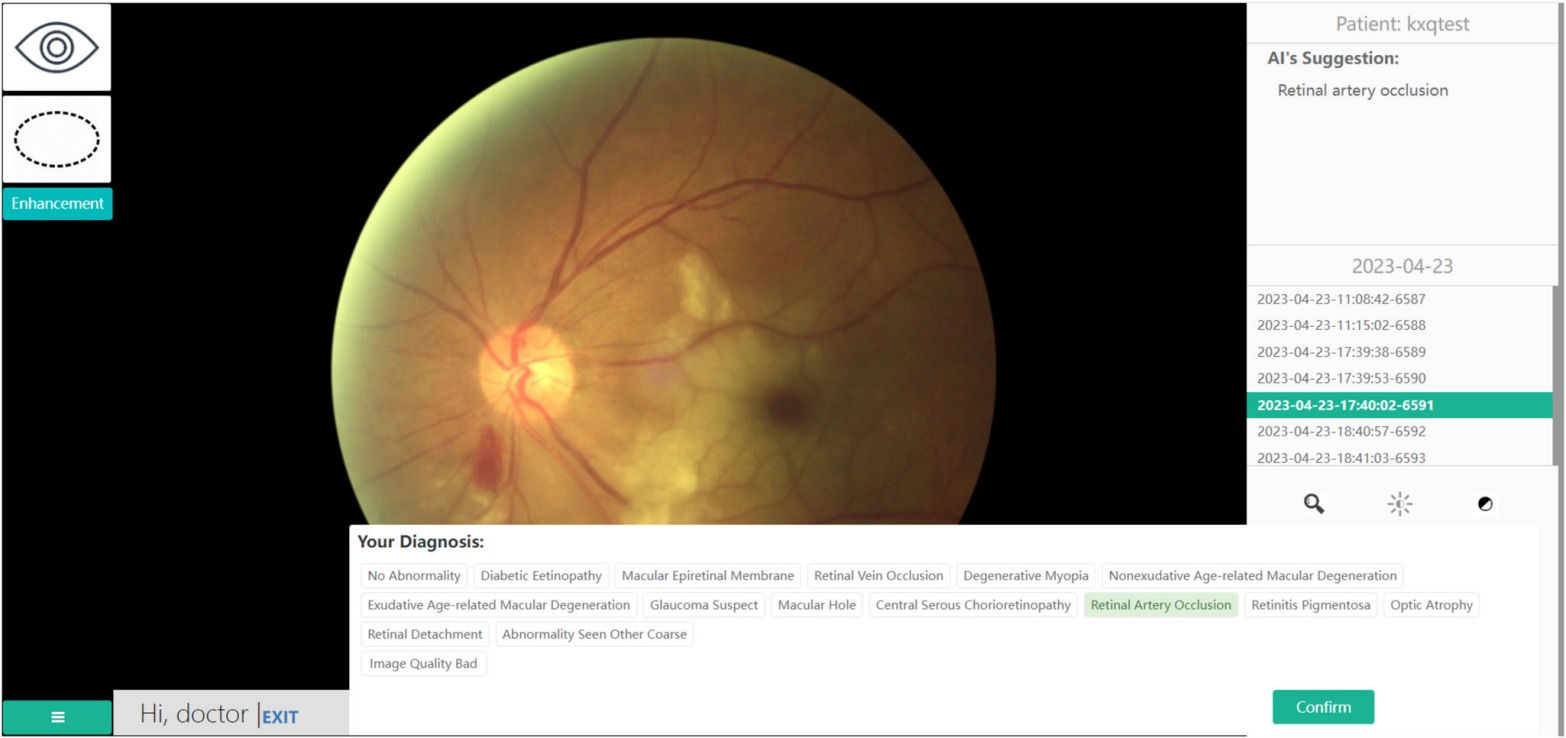
Dialog options of the DLS for assisting the junior ophthalmologists. The AI’s suggested label is presented at the upper right corner. The doctors could choose the disease label at the bottom.

Nine junior ophthalmologists, ophthalmic residents or attendings with clinical experience of 3 ~ 5 years of clinical experience, were selected from the five participating centers. They had not received subspecialty training and thus represented the diagnostic capacity of qualified general junior ophthalmologists. Before the formal annotation, they were trained on how to work with the system, on the labeling principles for multiple diseases and on the referral role of some specific diseases including DR and pathologic myopia. The diagnostic criteria of the included diseases and their clinical characteristics were not included in the training to maintain the real diagnostic capacity of junior ophthalmologists in clinical practice. After the training phase, each of the junior ophthalmologists was assigned to annotate portions of the datasets independently as the control group; specifically, each ophthalmologist annotated images obtained from the participating center they worked at. After a one-week washout period, they annotated the same groups of images, which had been randomly reordered and attached with labels previously annotated by the DLS, forming the test group. The dialog options of the DLS presented to the junior ophthalmologists for assistance is shown in Fig. 6. All the enrolled fundus images were also annotated by the DLS as the DLS group. Evaluation and comparison were conducted among the three groups.

### Outcomes measurements

#### Primary outcome

**Fig. 7:**
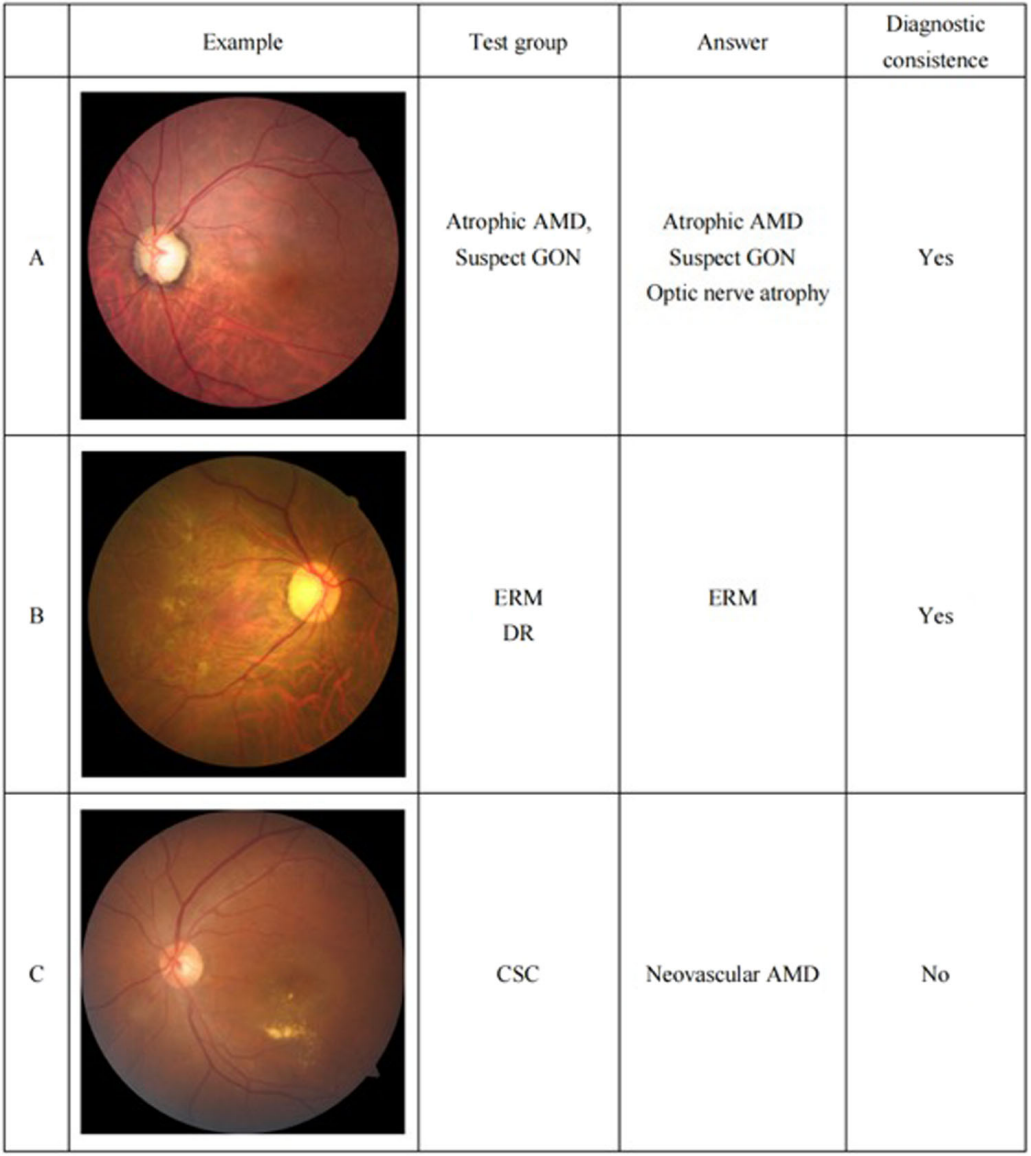
The illustration of consistency and inconsistency. Taking the three images as an example, if the test group correctly labels a portion of the standard annotation (A, B), the performance is considered as consistent. Otherwise, it is judged as inconsistent.

Considering that each image could be annotated with more than one label, we introduced a new indicator in this study, diagnostic consistency, as the primary outcome. The new metric refers to the percentage of the total images that had some labels matching the standard diagnosis, which means that the images could be labeled with other diseases together with the standard disease(s) or without some of the standard labels (as demonstrated in Fig. 7).

#### Secondary outcomes

Secondary measurements included the diagnostic accuracy of each image, which reflects the proportion of the images with all labels consistent with the standard diagnosis. The sensitivity, specificity and F1 score of each label were calculated for each group. The accuracy of the principal diagnosis was calculated in the test group and control group.

#### Post hoc analysis

First, we explored the junior ophthalmologists’ behaviors with the assistance of the DLS, especially, whether they tended to accept or overrule the AI’s suggestion. Second, to save manpower and simulate real application scenarios in clinical practice, we also analyzed the efficacy of a two-step protocol. The DLS was used as an initial filter in the first step. If the image was reported as “normal”, it did not proceed to the next step. Otherwise, the image was delivered to the junior ophthalmologists for final judgment. Metrics including diagnostic consistency and accuracy, sensitivity, specificity and F1 score were calculated and analyzed.

### Sample size calculation and statistical analysis

The DLS was developed for the detection of major fundus diseases and to help junior ophthalmologists reach higher diagnostic capacities. The study was designed as a superiority trial. The margin of superiority power was set to 0. We defined study success as the lower bound of the 95% CI of the difference in diagnostic consistency between the test group and control group being greater than 0. According to our preliminary experiments, the mean diagnostic consistency of junior ophthalmologists and associate professors was 55.8% and 68.4%, respectively. We assume that junior ophthalmologists could reach a comparable or even higher diagnostic consistency to that of associated professors with the assistance of the DLS system. Sample sizes were calculated based on the results of the preliminary experiments with a two-sided 5% Type 1 error, a 10% Type 2 error and 20% fall off. The calculated sample size was 940. Considering that some diseases are bilaterally involved, which can also affect the sample size calculation, and to ensure a sufficient sample for each disease as much as possible, we enlarged the total sample size to 1500 images (750 patients). The statistical assessment, including sample size calculation, was conducted using SPSS 22.0 (IBM Corp., Armonk, NY, USA). All results are presented as the mean ± SD with 95% CI. The Cochran‒Mantel‒Haenszel (CMH-χ2) test was applied for the comparison of diagnostic consistency. A P value below 0.05 was considered to indicate statistical significance. For the metric that did not coincide with a normal distribution, we compared the mean and median values.

### Reporting summary

Further information on research design is available in the Nature Research Reporting Summary linked to this article.

### Supplementary information

Supplementary Information

Reporting Summary

## Data Availability

Data available on request from the authors: The data that support the findings of this study are available from the corresponding author upon reasonable request.

## Code availability

The code or algorithm in this study can be accessed from the corresponding author upon reasonable request.
