# Supplementary material for: The performance of a deep learning system in assisting junior ophthalmologists in diagnosing 13 major fundus diseases: a prospective multi-center clinical trial
Source: NPJ Digit Med. 2024 Jan 11;7:8. doi: 10.1038/s41746-023-00991-9 (PMC10784504; doi:10.1038/s41746-023-00991-9)
Supplement: Supplementary file 1 — Supplementary Information [file 41746_2023_991_MOESM1_ESM.docx]

Supplementary Information

Table 1 Descriptions of the main features in the fundus images of the 13 selected fundus diseases

| Diseases | Standard diagnostic criteria |
| --- | --- |
| Referable diabetic retinopathy | ①Clear medical history of diabetes. ②The fundus images presented a diabetic retinopathy severity level of moderate nonproliferative diabetic retinopathy or worse, diabetic macular edema^1^. |
| Retinal vein occlusion | The fundus appearance presented with flame-shaped hemorrhage, dilation of the involved veins, cotton-wool spots, with or without papilledema^2^. |
| Retinal artery occlusion | The fundus appearance presented with local or extensive retinal pallor and edema alone or with attenuated retinal arteries, cheery-red spots at the macula area^3^. |
| Pathologic myopia | ①The myopic diopter should be over -6.0DS. ②The fundus images presented myopic maculopathy lesions in category 2-4 according to the META-PM (meta analyses of pathologic myopia) study classification^4^. |
| Retinitis pigmentosa | Fundus appearance including bone spicule pigmentations, thin retinal arteries and pallor of optic papilla^5^. |
| Retinal detachment | The elevation of the sensory retina presented as translucent membranoid structure with vessels on. This category included three major types of RD: rhegmatogenous, tractional and exudative. |
| Epiretinal membrane | A thin glistening membrane overlying the macula with or without retinal wrinkling6 |
| Atrophic age-related macular degeneration | Early, intermediate AMD^7^ and geographic atrophy |
| Neovascular age-related macular degeneration | Referring to neovascular AMD, which belongs to the late stage of AMD according to Age-Related Eye Disease Study (AREDS)^7^ |
| Macular hole | Stage 2-4 according to Gass’s classification of macular hole^8^. |
| Possible glaucomatous optic neuropathy | With cup disc ratio greater than 0.6 with or without corresponding retinal fiber layer defect, or asymmetric rim narrowing |
| Central serous chorioretinopathy | Halo like serous retinal detachment at the macular with the fovea involved |
| Optic nerve atrophy | Pallor of the optic nerve^9^ |
| Other abnormalities | Abnormal fundus not associated with any of 13 mentioned diseases. |

References

1. Ting DSW, Cheung CY, Lim G, et al. Development and Validation of a Deep Learning System for Diabetic Retinopathy and Related Eye Diseases Using Retinal Images From Multiethnic Populations With Diabetes. JAMA 2017,318(22):2211-2223.

2. Robinson MK and Halpern JI. Retinal vein occlusion. Am Fam Physician 1992,45(6):2661-2666.

3. Hayreh SS and Zimmerman MB. Fundus changes in central retinal artery occlusion. Retina 2007,27(3):276-289.

4. Ohno-Matsui K, Kawasaki R, Jonas JB, et al. International photographic classification and grading system for myopic maculopathy. Am J Ophthalmol 2015,159(5):877-883 e877.

5. Hartong DT, Berson EL and Dryja TP. Retinitis pigmentosa. Lancet 2006,368(9549):1795-1809.

6. Bu SC, Kuijer R, Li XR, Hooymans JM and Los LI. Idiopathic epiretinal membrane. Retina 2014,34(12):2317-2335.

7. Ferris FL, 3rd, Wilkinson CP, Bird A, et al. Clinical classification of age-related macular degeneration. Ophthalmology 2013,120(4):844-851.

8. Johnson RN and Gass JD. Idiopathic macular holes. Observations, stages of formation, and implications for surgical intervention. Ophthalmology 1988,95(7):917-924.

9. Biousse V and Newman NJ. Diagnosis and clinical features of common optic neuropathies. Lancet Neurol 2016,15(13):1355-1367.
