# Supplementary material for: The performance of a deep learning system in assisting junior ophthalmologists in diagnosing 13 major fundus diseases: a prospective multi-center clinical trial
Source: NPJ Digit Med. 2024 Jan 11;7:8. doi: 10.1038/s41746-023-00991-9 (PMC10784504; doi:10.1038/s41746-023-00991-9)
Supplement: Supplementary file 2 — Reporting Summary [file 41746_2023_991_MOESM2_ESM.pdf]

nature portfolio

Corresponding author(s): CYX NPJDIGITALMED-06535

Last updated by author(s): Aug 4, 2023

# Reporting Summary

Nature Portfolio wishes to improve the reproducibility of the work that we publish. This form provides structure for consistency and transparency in reporting. For further information on Nature Portfolio policies, see our Editorial Policies and the Editorial Policy Checklist.

## Statistics

For all statistical analyses, confirm that the following items are present in the figure legend, table legend, main text, or Methods section.

| n/a | Confirmed | |
|---|---|---|
| ☐ | ☒ | The exact sample size (*n*) for each experimental group/condition, given as a discrete number and unit of measurement |
| ☐ | ☒ | A statement on whether measurements were taken from distinct samples or whether the same sample was measured repeatedly |
| ☐ | ☒ | The statistical test(s) used AND whether they are one- or two-sided<br>*Only common tests should be described solely by name; describe more complex techniques in the Methods section.* |
| ☐ | ☒ | A description of all covariates tested |
| ☐ | ☒ | A description of any assumptions or corrections, such as tests of normality and adjustment for multiple comparisons |
| ☐ | ☒ | A full description of the statistical parameters including central tendency (e.g. means) or other basic estimates (e.g. regression coefficient) AND variation (e.g. standard deviation) or associated estimates of uncertainty (e.g. confidence intervals) |
| ☐ | ☒ | For null hypothesis testing, the test statistic (e.g. *F*, *t*, *r*) with confidence intervals, effect sizes, degrees of freedom and *P* value noted<br>*Give P values as exact values whenever suitable.* |
| ☒ | ☐ | For Bayesian analysis, information on the choice of priors and Markov chain Monte Carlo settings |
| ☒ | ☐ | For hierarchical and complex designs, identification of the appropriate level for tests and full reporting of outcomes |
| ☒ | ☐ | Estimates of effect sizes (e.g. Cohen's *d*, Pearson's *r*), indicating how they were calculated |

*Our web collection on statistics for biologists contains articles on many of the points above.*

## Software and code

Policy information about availability of computer code

| | |
|---|---|
| Data collection | no software was used for data collection |
| Data analysis | SPSS 22.0 was used for data analysis in this study |

For manuscripts utilizing custom algorithms or software that are central to the research but not yet described in published literature, software must be made available to editors and reviewers. We strongly encourage code deposition in a community repository (e.g. GitHub). See the Nature Portfolio guidelines for submitting code & software for further information.

## Data

Policy information about availability of data

All manuscripts must include a data availability statement. This statement should provide the following information, where applicable:

- Accession codes, unique identifiers, or web links for publicly available datasets
- A description of any restrictions on data availability
- For clinical datasets or third party data, please ensure that the statement adheres to our policy

Data available on request from the authors: The data that support the findings of this study are available from the corresponding author upon reasonable request.

## Research involving human participants, their data, or biological material

Policy information about studies with human participants or human data. See also policy information about sex, gender (identity/presentation), and sexual orientation and race, ethnicity and racism.

| | |
|---|---|
| Reporting on sex and gender | In this report, we collected sex information as part of the results of demography. We didn't provide sex based analysis, because most of the diseases involved presented no sex associated trends. The reporting in the manuscript is as follows (Line 273-275):<br>The mean (SD) age of the patients was 51.7 (14.7) years (range, 18~75), and 324 (43.3%) of them were male. All participants were Chinese Han patients. |
| Reporting on race, ethnicity, or other socially relevant groupings | The patients enrolled in our study are all Chinese Han people. The information was attained from their valid identity documents. This is not a confounding variables for data analysis, so we didn't provide additional discussion. The reports in the manuscript is as follows:<br>All participants were Chinese Han patients. |
| Population characteristics | We collected and described the population characteristics including age, sex , race and medical history. The report in the manuscript is as follows:<br>The mean (SD) age of the patients was 51.7 (14.7) years (range, 18~75), and 324 (43.3%) of them were male. All participants were Chinese Han patients. Regarding the medical history of the patients, 152 (20.3%), 216 (28.9%) and 104 (13.9%) patients reported having diabetic mellites, hypertension and hyperlipemia, respectively. |
| Recruitment | The participants are recruited by doctors at the outpatient survives. They select patients according to inclusion and exclusion criteria. However, patients from the outpatient clinic may present with much higher frequency of abnormalities than real screening scenario. This may bring some bias to the report about the distribution of diseases. |
| Ethics oversight | The current study complied with the Declaration of Helsinki and was approved by the ethics committee of each participanting center. |

Note that full information on the approval of the study protocol must also be provided in the manuscript.

# Field-specific reporting

Please select the one below that is the best fit for your research. If you are not sure, read the appropriate sections before making your selection.

☒ Life sciences ☐ Behavioural & social sciences ☐ Ecological, evolutionary & environmental sciences

For a reference copy of the document with all sections, see nature.com/documents/nr-reporting-summary-flat.pdf

# Life sciences study design

All studies must disclose on these points even when the disclosure is negative.

| | |
|---|---|
| Sample size | The DLS was developed for the detection of major fundus diseases and to help junior ophthalmologists reach higher diagnostic capacities. Therefore, in this study, we set the prespecified superiority endpoint as the achievement of diagnostic consistency among the junior ophthalmologists with DLS assistance comparable to that of associate professors. According to our preliminary experiments, the mean diagnostic consistency of the junior ophthalmologists and associate professors was 55.8% and 68.4%, respectively. Sample sizes for these hypotheses were calculated for at least 68% power, a two-sided 5% Type 1 error, a 10% Type 2 error and 20% fall off. The calculated sample size was 940. Considering that some diseases are bilaterally involved which can also affect the sample size calculation, and to ensure a sufficient sample for each disease as much as possible, we enlarged the total sample size to 1500 images (750 patients). |
| Data exclusions | A total of 750 patients were screened from five participating hospitals, and 748 of them completed all procedures. After the standard annotation, three images were excluded due to unsatisfactory image quality. |
| Replication | As a clinical trial, the findings were not replicated. |
| Randomization | This is a self-control clinical trial. Randomization was not applied in this study. |
| Blinding | This is a self-control clinical trial. Blingding was not applied in this study. |

# Reporting for specific materials, systems and methods

We require information from authors about some types of materials, experimental systems and methods used in many studies. Here, indicate whether each material, system or method listed is relevant to your study. If you are not sure if a list item applies to your research, read the appropriate section before selecting a response.

## Materials & experimental systems

| n/a | Involved in the study | |
|---|---|---|
| ☒ | ☐ | Antibodies |
| ☒ | ☐ | Eukaryotic cell lines |
| ☒ | ☐ | Palaeontology and archaeology |
| ☒ | ☐ | Animals and other organisms |
| ☐ | ☒ | Clinical data |
| ☒ | ☐ | Dual use research of concern |
| ☒ | ☐ | Plants |

## Methods

| n/a | Involved in the study | |
|---|---|---|
| ☒ | ☐ | ChIP-seq |
| ☒ | ☐ | Flow cytometry |
| ☒ | ☐ | MRI-based neuroimaging |

# Clinical data

Policy information about clinical studies

All manuscripts should comply with the ICMJE guidelines for publication of clinical research and a completed CONSORT checklist must be included with all submissions.

Clinical trial registration

It is registered on ClinicalTrials.gov with registration number NCT04723160.

Study protocol

https://clinicaltrials.gov/study/NCT04723160?term=NCT04723160&rank=1

Data collection

A total of 750 participants were prospectively screened in this observational study at the five centers from August 2020 to January 2021.

Outcomes

Primary outcome: Considering that each image could be annotated with more than one label, we introduced a new indicator in this study, diagnostic consistency, as the primary outcome. The new metric refers to the percentage of the total images that had some labels matching the standard diagnosis, which means that the images could be labeled with other diseases together with the standard disease(s) or without some of the standard labels (as demonstrated in Figure 4).

Secondary outcomes: Secondary measurements included the diagnostic accuracy of each image, which reflects the proportion of all labels that were consistent with the standard diagnosis. The sensitivity, specificity and F1 score of each label were calculated for each group. The accuracy of the principal diagnosis was calculated in the test group and control group.
